# Non-linear Dose Response of Lymphocyte Cell Lines to Microtubule Inhibitors

**DOI:** 10.3389/fphar.2019.00436

**Published:** 2019-04-24

**Authors:** Daria M. Potashnikova, Aleena A. Saidova, Anna V. Tvorogova, Eugene V. Sheval, Ivan A. Vorobjev

**Affiliations:** ^1^Department of Cell Biology and Histology, School of Biology, M.V. Lomonosov Moscow State University, Moscow, Russia; ^2^Laboratory of Atherothrombosis, Moscow State University of Medicine and Dentistry, Moscow, Russia; ^3^Department of Cell Biotechnology, Center of Experimental Embryology and Reproductive Biotechnology, Moscow, Russia; ^4^A.N. Belozersky Institute of Physico-Chemical Biology, M.V. Lomonosov Moscow State University, Moscow, Russia; ^5^Department of Biology, School of Science and Technology, Nazarbayev University, Astana, Kazakhstan

**Keywords:** cell cycle, cell death, microtubule inhibitors, paclitaxel, nocodazole, vinorelbine, B-lymphocytes, RPMI8866

## Abstract

Microtubule (MT) inhibitors show anti-cancer activity in a wide range of tumors *in vitro* and demonstrate high clinical efficacy. To date they are routinely included into many chemotherapeutic regimens. While the mechanisms of MT inhibitors’ interactions with tubulin have been well-established, the relationship between their concentration and effect on neoplastic cells is not completely understood. The common notion is that tumor cells are most vulnerable during division and all MT inhibitors block them in mitosis and induce mitotic checkpoint-associated cell death. At the same time multiple evidence of more subtle effects of lower doses of MT inhibitors on cell physiology exist. The extent of efficacy of the low-dose MT inhibitor treatment and the mechanisms of resulting cell death currently present a critical issue in oncology. The prospect of MT inhibitor dose reduction is promising as protocols at higher concentration have multiple side effects. We assessed cell cycle changes and cell death induced by MT inhibitors (paclitaxel, nocodazole, and vinorelbine) on human lymphoid B-cell lines in a broad concentration range. All inhibitors had similar accumulation effects and demonstrated “trigger” concentrations that induce cell accumulation in G2/M phase. Concentrations slightly below the “trigger” promoted cell accumulation in sub-G1 phase. Multi-label analysis of live cells showed that the sub-G1 population is heterogeneous and may include cells that are still viable after 24 h of treatment. Effects observed were similar for cells expressing Tat-protein. Thus cell cycle progression and cell death are differentially affected by high and low MT inhibitor concentrations.

## Introduction

The importance of microtubule (MT) dynamics for cell motility (Liao et al., 1995), migration (Kaverina and Straube, 2011), and division (Jordan et al., 1993) has been well-established. The multi-aspect effect of suppressed microtubule dynamics makes MT inhibitors an important part of most anti-cancer chemotherapeutic regimens by inhibiting tumor growth (antimitotic effect) and metastasis (antimigration effect) (Dumontet and Jordan, 2010).

Microtubule inhibitors encompass several classes of compounds with varying mechanisms of interactions with tubulin (Perez, 2009) that disrupt MT dynamics by either stabilizing or de-stabilizing them. MT inhibitors of the first generation widely used in clinics (taxanes, vinca alkaloid derivatives) have certain drawbacks, including neurotoxic effects (Windebank, 1999), neutropenia (Donehower and Rowinsky, 1993) and loss of efficacy against advanced forms of some cancers (Sève et al., 2008). Clinical limitations in treatment of solid tumors and hematologic malignancies prompted further research for analogs with improved clinical characteristics, recently introducing vinflunine, epothilones, indibulin, and many other (Bennouna et al., 2008; Cortes and Vidal, 2012).

Varying effects of high and low MT inhibitor concentrations on different aspects of cell physiology have been described for a number of cell models (Grigoriev et al., 1999; Yang et al., 2010). The first meta-analysis focused on efficacy and toxicity of low-dose versus conventional-dose chemotherapies has been published recently (Xie et al., 2017) and provided data on low-dose chemotherapy advantages.

Microtubule inhibitors are integrated into a variety of chemotherapy schemes for B-cell malignancies, including the curative regimens of non-Hodgkin lymphomas (Bates et al., 2016). Their anti-cancer effects extend beyond their ability arrest mitosis and include their potential to induce apoptosis at all cell cycle phases (Bates and Eastman, 2017). The physiological consequences of MT dynamics inhibition are still poorly understood; the elucidation of its pleiotropic effects on cell death and cell cycle will provide novel therapeutic strategies. In this study we sought to determine the lowest efficient concentration of several MT inhibitors and tested their dose-dependent effects on cell death and mitotic arrest on B-cell lines RPMI8866 and its Tat-expressing modification RPMI8866-Tat-GFP. B-cell lymphomas (Burkitt lymphoma, DLBCL, and B-CLL) are one of the most common comorbid conditions for HIV-infected patients (Gopal et al., 2013). B-cell oncogenesis in HIV patients is related to Tat viral protein that enters B-lymphocytes, acts as a transcription factor in oncogenic cascades (Vendrame et al., 2014; Musinova et al., 2016) but also has cytoplasmic targets including MTs (de Mareuil et al., 2005). To our knowledge, no current research describes the effect of MT inhibitors on Tat-expressing B-cells.

## Materials and Methods

### Cell Lines and Microtubule Inhibitors

Human B-cell [RPMI8866 and Tat-GFP expressing RPMI8866 (RPMI8866-Tat-GFP)] and T-cell (Jurkat) lines were used in the study. Cells were maintained in EX-CELL Medium (SAFC Biosciences, United States) supplemented with L-glutamine and 10% FBS (Paneco, Russia). The effects of three MT inhibitors on cell cycle and cell death were evaluated in the study: paclitaxel (taxol; Sigma, United States), nocodazole (Biochem, United States) and vinorelbine (Sigma, United States). MT inhibitors were added to the medium 24 h prior to analysis at final concentrations of 3; 10; 30; 100; 300; and 1000 nM.

### Fluorescence Staining

Propidium iodide for DNA staining: The protocol for cell cycle analysis in suspension cells was published previously (Potashnikova et al., 2018).

Anti-caspase 3 immunostaining kit: PE-conjugated antibodies against active caspase 3 (BD, United States) were used to assess percentages of apoptotic cells. Cell fixation, permeabilization and staining were performed using supplied buffers based on the manufacturer’s instructions.

Multi-label cell cycle and apoptosis staining: live cell suspensions of RPMI8866 and RPMI8866-Tat-GFP were simultaneously stained with tetramethyl-rhodamine ethyl ester (TMRE) for membrane potential, Hoechst33342 for DNA and annexin V-Alexa Fluor 647 (all Thermo Fisher Scientific, United States) for phosphatidylserine externalization. RPMI8866 cells were also stained with CellEvent fluorescent caspase 3/7 substrate (Thermo Fisher Scientific, United States): RPMI8866-Tat-GFP cells were not stained with CellEvent to avoid fluorescence signal conflict. The staining protocol was published previously (Vorobjev and Barteneva, 2016).

### Flow Cytometry

Cells were analyzed using a FACSAria SORP cell sorter at Ex.407 nm/Em.425–475 nm for Hoechst33342, Ex.488 nm/Em.515–535 nm for CellEvent, Ex.561 nm/Em.575–590 nm for TMRE, PI and anti-caspase3-PE and Ex.640 nm/Em.663–677 nm for annexin V-Alexa Fluor 647. Flow cytometry data were acquired and analyzed using FACS Diva v.6.1.3 software (BD, United States). Each measurement was performed at least in triplicate.

### Fluorescence Microscopy

Cell suspensions simultaneously stained with TMRE, Hoechst33342, CellEvent and annexin V-Alexa Fluor 647 were analyzed using a Nikon Ti Eclipse microscope under PlanApo 60×/1.4 objective. Images were recorded by Hamamatsu ORCA FLASH2 digital camera, using the following filter sets: Ex.450–490 nm/Em.510–540 nm; Ex.340–380 nm/Em.435–485 nm; Ex.530–560 nm/Em.573–640 nm and Ex.595–645 nm/Em.665–715 nm.

### Statistical Analysis

Data from biological repeats were calculated as mean and standard deviation and plotted in GraphPad Prism software (United States). Unpaired *t*-test (95% confidence interval) was used as significance test.

### Electron Microscopy

Cells were fixed in 4% glutaraldehyde (Ted Pella, United States) in 0.1 M Sørensen phosphate buffer for 2 h, post-fixed with 1% osmium tetroxide (Sigma, United States) for 1 h, dehydrated in ethanol and propylene oxide and embedded in Spi-pon 812 epoxy resin (SPI Inc., United States). Ultrathin sections were cut using an LKB III ultramicrotome (LKB), stained with uranyl acetate and lead citrate, and photographed using a JEM-1400 electron microscope (Jeol, Japan)^1^.

## Results

### The Mitostatic Response to Various MT Inhibitor Concentrations Is Non-linear

**FIGURE 1:**
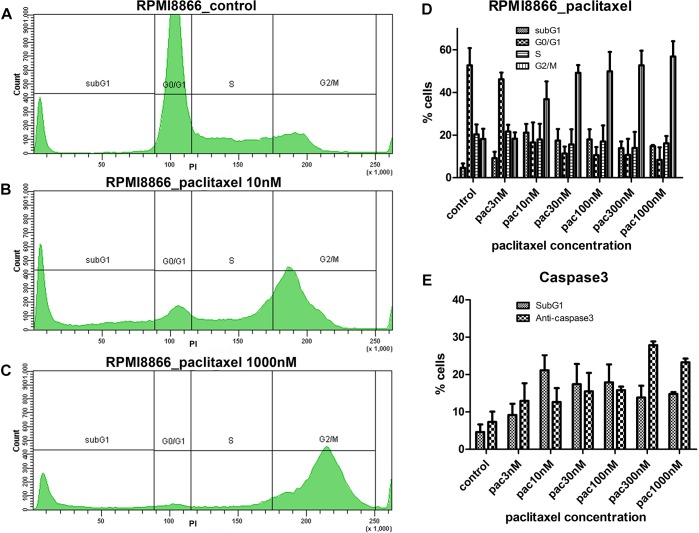
RPMI8866 B-lymphocytes untreated (control) and treated with paclitaxel (concentration range 3, 10, 30, 100, 300, and 1000 nM) for 24 h. **(A–C)** DNA content curves in individual samples assessed by flow cytometry after propidium iodide staining and linear gates set to determine sub-G1, G0/G1, S, and G2/M cell cycle populations. 50 000 events were analyzed per sample. **(A)** Normal cell cycle distribution in control cells; G2/M comprises 19% cells, sub-G1 comprises 5% cells. **(B)** G2/M accumulation after 10 nM paclitaxel treatment; G2/M comprises 38% cells, sub-G1 comprises 25% cells. **(C)** G2/M accumulation after 1000 nM paclitaxel treatment; G2/M comprises 63% cells, sub-G1 comprises 15% cells. **(D)** Data from biological repeats on sub-G1, G0/G1, S, and G2/M population distributions were presented as mean percentages with *SD* on a histogram. Each measurement was performed at least in triplicate. **(E)** Miscorrelation of sub-G1 population numbers and caspase 3-positive cell numbers after paclitaxel treatment. The largest sub-G1 peak is observed at 10 nM paclitaxel while the largest caspase 3-positive population is observed at 300 nM paclitaxel.

We tested the mitostatic effect of three common stabilizing (paclitaxel) and depolymerizing (nocodazole, vinorelbine) MT inhibitors, on RPMI8866, RPMI8866-Tat-GFP (B-lymphocytes) and Jurkat (T-lymphocytes) cell lines. Linear gates were set to determine cell percentages at different cell cycle stages on DNA content curves (Figure 1A–C).

Microtubule inhibitors uniformly prompted cell accumulation in G2/M in a non-linear fashion: we found “trigger” concentrations sufficient to accumulate cells in G2/M phase that fell into 10–100 nM range for all inhibitors and cell lines. Concentrations below the “trigger” retained cell cycle distribution close to normal. For example, for 3 nM paclitaxel we observed 46% cells in G0/G1, 22% cells in S, and 18% in G2/M for RPMI8866 cells compared to 53% cells in G0/G1, 20% cells in S, and 18% in G2/M in control (Figure 1D). Concentrations above the “trigger” increased the G2/M population peak with a subsequent decrease of the G1 peak (Figure 1B,C and Supplementary Figure S1). Similar response patterns were achieved for every MT inhibitor; however, paclitaxel graphs were chosen as most representative.

### The Sub-G1 Population on DNA Content Curves Likely Represents Apoptotic Cells but Its Percentage Does Not Correlate With Percentages of Caspase-3 Positive Cells

The number of cells with sub-G1 DNA content increased significantly in every MT inhibitor concentration compared to untreated control (*p* < 0.05, unpaired *t*-test) (Figure 1A–C and Supplementary Figure S1). Hypodiploid amounts of DNA can be explained by nucleic acid degradation in late apoptosis. Intriguingly, the largest sub-G1 populations did not correspond to the highest MT inhibitor concentrations. Sub-G1 cell percentages did not significantly differ at “trigger,” sub-“trigger” and high MT inhibitor concentrations. The only significant difference was obtained for paclitaxel: the highest percentage of cells in sub-G1 (21%) was present in 10 nM paclitaxel; that was significantly increased compared to 3, 300, and 1000 nM paclitaxel and insignificantly different from 30 to 100 nM paclitaxel (*p* < 0.05, unpaired *t*-test). To assess apoptosis levels, cells were immunostained for active caspase 3. Lack of correlation between sub-G1 and caspase 3-positive cell percentages was observed (Figure 1E) as the number of caspase 3-positive cells at 10 nM paclitaxel (13%) was similar to the numbers of caspase 3-positive cells in the range of 3–100 nM paclitaxel. To further investigate the apparent contradiction between the results obtained by DNA content analysis and anti-caspase 3 staining a novel protocol for live cell analysis was developed and tested on B-lymphocytes.

### Live Cell Staining With Hoechst33342 Provides Similar Results to PI Staining and Allows a Simultaneous Multi-Parameter Apoptosis Assessment

**FIGURE 2:**
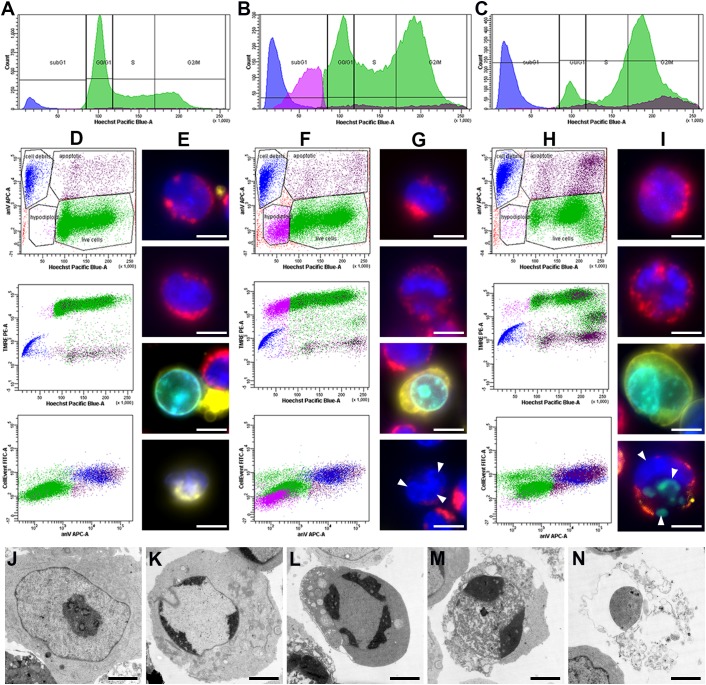
RPMI8866 B-lymphocytes untreated (control) and treated with paclitaxel (10 and 1000 nM) for 24 h. **(A–C)** DNA content curves in individual samples assessed by flow cytometry after Hoechst33342 staining and linear gates set to determine sub-G1, G0/G1, S, and G2/M cell cycle populations [colored subpopulations correspond to live cells (green), apoptotic cells (brown), hypodiploid cells (purple) and cell debris (blue) as gated on dot plots **(D–F)**]. 50 000 events were analyzed per sample. **(A)** Normal cell cycle distribution in control cells; G2/M comprises 18% cells, sub-G1 comprises 6% cells. **(B)** G2/M accumulation after 10 nM paclitaxel treatment; G2/M comprises 30% cells, sub-G1 comprises 26% cells. **(C)** G2/M accumulation after 1000 nM paclitaxel treatment; G2/M comprises 49% cells, sub-G1 comprises 19% cells. **(D,F,H)** Dot plots representing simultaneous staining of RPMI8866 B-lymphocytes with Hoechst33342, TMRE, annexin V-Alexa Fluor 647, and CellEvent. Region gates are set to determine live cells (green), apoptotic cells (brown), hypodiploid cells (purple) and cell debris (blue) based on Hoechst33342 vs. annexin V staining. **(D)** Normal apoptosis rate in control cells. Region gates are set to live cells (green) – 89.4%, apoptotic cells (brown) – 3.2%, hypodiploid cells (purple) – 0.6% and cell debris (blue) – 6.8% based on Hoechst33342 vs. annexin V staining. The colors of the gated populations remain the same in the TMRE vs. Hoechst33342 and CellEvent vs. annexin V graphs. **(F)** G2/M and sub-G1 accumulation after 10 nM paclitaxel treatment. Region gates are set to live cells (green) – 69.0%, apoptotic cells (brown) – 4.9%, hypodiploid cells (purple) – 13.5% and cell debris (blue) – 12.6% based on Hoechst33342 vs. annexin V staining. The colors of the gated populations remain the same in the TMRE vs. Hoechst33342 and CellEvent vs. annexin V graphs. **(H)** G2/M accumulation after 1000 nM paclitaxel treatment. Region gates are set to live cells (green) – 67.0%, apoptotic cells (brown) – 13.3%, hypodiploid cells (purple) – 1.6% and cell debris (blue) – 18.1% based on Hoechst33342 vs. annexin V staining. The colors of the gated populations remain the same in the TMRE vs. Hoechst33342 and CellEvent vs. annexin V graphs. **(E,G,I)** Fluorescence images of cells untreated (control) and treated with paclitaxel (10 and 1000 nM) for 24 h. Simultaneous staining of RPMI8866 B-lymphocytes with Hoechst33342 (blue), TMRE (red), annexin V-Alexa 647 (yellow), and CellEvent (green). Scale bar – 10 μm. **(E)** Control cells; top two images correspond to live cells with bright nuclear Hoechst33342 and mitochondrial TMRE fluorescence, bottom two images correspond to an apoptotic cell with CellEvent staining in the nucleus and surface annexin V and cell debris with a dim nucleus and surface annexin V. **(G)** 10 nM paclitaxel treatment; top two images correspond to live cells with bright nuclear Hoechst33342 and mitochondrial TMRE fluorescence, third image corresponds to an apoptotic cell with CellEvent staining in the nucleus and surface annexin V, bottom image corresponds to aneuploid (hypodiploid) cell with dim mitochondrial TMRE fluorescence and micronuclei. White arrowheads indicate micronuclei. **(I)** 1000 nM paclitaxel treatment; top two images correspond to live cells with bright nuclear Hoechst33342 and mitochondrial TMRE fluorescence, bottom two images correspond to apoptotic cells with CellEvent staining in the nucleus and surface annexin V with/without micronuclei. White arrowheads indicate micronuclei. **(J–N)** Electron microscopy of cells at various stages of apoptosis after 10 nM paclitaxel treatment. Scale bar – 2 μm. **(J)** Normal cell with typical RPMI8866 lymphocyte morphology. **(K)** Chromatin marginalization. **(L)** Decrease of cell volume and condensation of cytoplasm (cell shrinkage). **(M)** Early stage of cell destruction (secondary necrosis). **(N)** Cellular debris inside which the residual chromatin and cytoplasmic organelles can be distinguished.

Live cell suspensions of RPMI8866 and RPMI8866-Tat-GFP were simultaneously stained with TMRE, Hoechst33342, CellEvent fluorescent caspase 3/7 substrate and annexin V-Alexa Fluor 647. Combined DNA content and apoptotic marker cell labeling was visualized using flow cytometry and fluorescence microscopy. Hoechst33342-based DNA content analysis provided a dose response pattern similar to PI staining with G2/M peak shift at “trigger” concentrations (Supplementary Figure S2). Dying cells exhibit the typical pattern of apoptotic events (Vorobjev and Barteneva, 2015): the gradual TMRE fluorescence decrease corresponding to mitochondrial membrane potential (MMP) loss, CellEvent fluorescence corresponding to caspase 3 activation and annexin V surface staining corresponding to phosphatidylserine externalization (Figure 2D–I). This result was confirmed by electron microscopy (Figure 2J–N): multiple apoptotic cells were detected in the paclitaxel-treated specimen. Early stages were characterized by chromatin marginalization; at later stages, cell volume decreased markedly, accompanied by cytoplasm condensation. Dead cells had destroyed plasma membranes and cytoplasmic organelles, but inside them the residuals of nuclei were still distinguishable.

Sub-G1 cell accumulation was observed at various rates upon MT inhibitor treatment and was most prominent in 10 nM paclitaxel (26%) (Figure 2A–C). The sub-G1 population was heterogeneous and contained cell debris with apoptotic markers and low MMP and cells with no apoptotic markers, hypodiploid DNA content and a two-fold decreased MMP compared to live cells with normal ploidy. Median TMRE fluorescence intensity was 976 a.u. for cell debris, 22380 a.u. for hypodiploid cells and 48265 a.u. for normal live cells (Figure 2F–G). Differences were statistically significant (*p* < 0.05). Fluorescence microscopy revealed live cells, apoptotic cells, cell debris and a fraction of small-sized live cells, often with micronuclei and dim mitochondria, in all MT inhibitor-treated specimens (Supplementary Figure S3).

## Discussion

It was shown that MT inhibitor concentrations sufficient for cell motility suppression can be lower than those needed for mitotic arrest (Kapoor and Panda, 2012; Molina et al., 2013). One of the thrilling questions is whether cytotoxic effects can be exerted at low concentrations of MT inhibitors. To answer this, we opted for a multi-parameter simultaneous cell cycle and cell death assessment. The proposed approach was derived from the two protocols described previously (Vorobjev and Barteneva, 2016; Potashnikova et al., 2018). It provides an easy and robust way to separately estimate the cytotoxic and mitostatic effects of microtubule inhibitors. The staining is suitable for high-throughput flow cytometry and imaging flow cytometry, as well as for microscopic evaluation of apoptosis kinetics in heterogeneous cell populations after chemotherapeutic treatment.

Using such an approach we described the non-linear dose response to an array of MT inhibitors, which seems to be universal. The “trigger” concentrations fall between 10***–***30 nM for paclitaxel, 30***–***100 nM for nocodazole and 10***–***30 nM for vinorelbine. Increasing dosages do not significantly alter G2/M accumulation. Despite the controversial data on B-cell responses to various types of MT inhibitors (Beswick et al., 2006; Frezzato et al., 2014) in our experiments the pattern of response to all compounds was similar for all cell lines. Our data on cell cycle accumulation and cell death percentages after MT inhibitor treatment correspond to the published data on adherent cells (Di Cesare et al., 2017).

Cells accumulated in G2/M phase die via apoptosis, exhibiting mitochondrial membrane potential (MMP) decrease, caspase activation and phosphatidylserine externalization. A sub-G1 population on DNA content curves includes late apoptotic cells and cell debris. However, at low paclitaxel concentrations this population also includes particularly large numbers of cells with MMP decreased only two-fold compared to normal cells and no markers of apoptosis. This explains the miscorrelation between sub-G1 and caspase 3-positive cell percentages observed in a range of paclitaxel concentrations. Such addition biases the interpretation of sub-G1 population as homogeneous and invariably apoptotic. Sub-G1 cells were described in other cell models as potentially non-viable products of aberrant mitosis (Demidenko et al., 2008) resulting from MT inhibition. Using fluorescence microscopy we visualized cells with smaller nuclei, often with micronuclei, and dim TMRE staining that fit the description of such cells. While their viability has to be further investigated, they present a distinctly different pattern of response to MT inhibitors compared to standard G2/M-accumulating cells that has to be accounted for.

Interestingly, MT inhibitors had the same effect on Tat-expressing B-lymphocytes (Supplementary Figures S1, S2). Despite some evidence of Tat protein interactions with microtubules (de Mareuil et al., 2005) RPMI8866-Tat-GFP cells do not differ from normal RPMI8866 lymphocytes in their MT inhibitor responses. The relevance of our findings for HIV-associated non-Hodgkin’s lymphoma model is important for treating such patients.

Thus cell cycle arrest and cell death may be differentially affected by high and low doses of MT inhibitors which has a big therapeutic potential in oncology.

## Author Contributions

DP and AS conceived and performed the experiments, analyzed the data, and wrote the manuscript. AT performed the experiments and analyzed the data. ES and IV conceived and performed the experiments, analyzed the data, and edited the manuscript.

## Conflict of Interest Statement

The authors declare that the research was conducted in the absence of any commercial or financial relationships that could be construed as a potential conflict of interest.

## Footnotes

1 For Original Research articles, please note that the Material and Methods section can be placed in any of the following ways: before Results, before Discussion or after Discussion.

## Supplementary Material

FIGURE S1 Lymphocytes treated with MT inhibitors (concentration range 3, 10, 30, 100, 300, and 1000 nM) for 24 h and stained for DNA content with propidium iodide. Data from biological repeats on sub-G1, G0/G1, S, and G2/M population distributions presented as mean percentages with *SD* on a histogram. Graphs in columns represent RPMI8866 B-lymphocytes, RPMI8866-Tat-GFP B-lymphocytes and Jurkat T-lymphocytes, respectively. Graphs in rows represent paclitaxel, nocodazole and vinorelbine, respectively. Jurkat cells were previously known to respond to MT inhibitors and were therefore used as control.

FIGURE S2 Lymphocytes treated with MT inhibitors (concentration range 3, 10, 30, 100, 300, and 1000 nM) for 24 h and stained for DNA content with Hoechst33342. Data from biological repeats on sub-G1, G0/G1, S, and G2/M population distributions presented as mean percentages with *SD* on a histogram. Graphs in columns represent RPMI8866 B-lymphocytes and RPMI8866-Tat-GFP B-lymphocytes, respectively. Graphs in rows represent paclitaxel, nocodazole and vinorelbine, respectively.

FIGURE S3 Fluorescence image galleries of RPMI8866 B-lymphocytes treated with a low dose of MT inhibitor 10 nM paclitaxel for 24 h. Simultaneous staining with Hoechst33342 (blue), TMRE (red), annexin V-Alexa 647 (yellow), and CellEvent (green). Scale bar – 10 μm. **(A)** Live cells with normal morphology have bright round nuclei, bright mitochondrial TMRE fluorescence and bear no apoptotic markers. **(B)** Apoptotic cells have TMRE-negative mitochondria, CellEvent caspase substrate staining co-localized with nuclear staining and surface-bound annexin V indicating phosphatidylserine externalization. **(C)** Cell debris and late apoptotic cells have smaller size, irregular shape, TMRE-negative mitochondria, deformed nuclei, often with CellEvent staining, and surface-bound annexin V indicating phosphatidylserine externalization. **(D)** small-sized cells with small nuclei, micronuclei, few TMRE-dim mitochondria, and no apoptotic markers. White arrowheads indicate micronuclei.
