# Supplementary figures and images for: Non-linear Dose Response of Lymphocyte Cell Lines to Microtubule Inhibitors
Source: Front Pharmacol. 2019 Apr 24;10:436. doi: 10.3389/fphar.2019.00436 (PMC6491834; doi:10.3389/fphar.2019.00436)

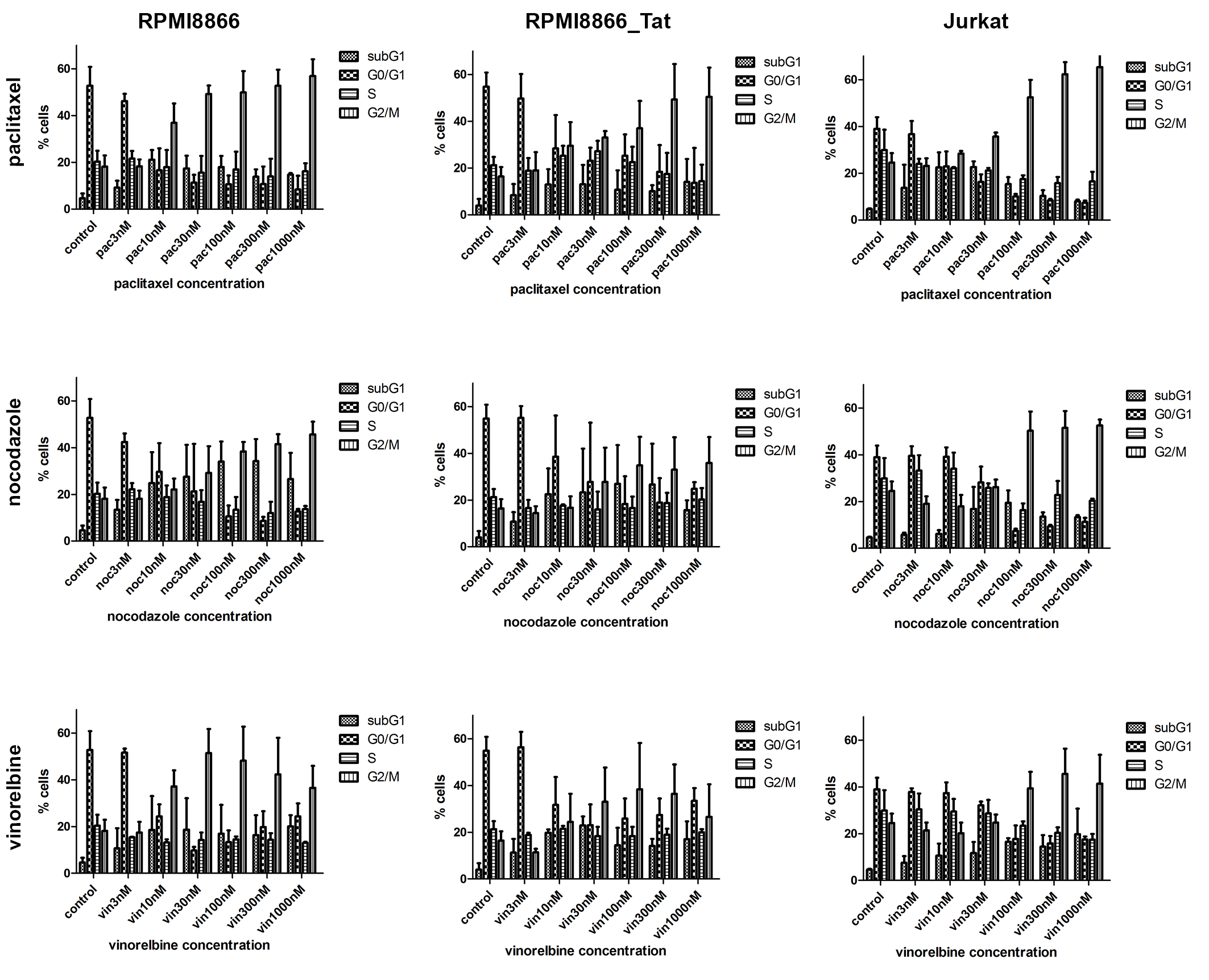

Supplement: FIGURE S1 — Lymphocytes treated with MT inhibitors (concentration range 3, 10, 30, 100, 300, and 1000 nM) for 24 h and stained for DNA content with propidium iodide. Data from biological repeats on sub-G1, G0/G1, S, and G2/M population distributions presented as mean percentages with SD on a histogram. Graphs in columns represent RPMI8866 B-lymphocytes, RPMI8866-Tat-GFP B-lymphocytes and Jurkat T-lymphocytes, respectively. Graphs in rows represent paclitaxel, nocodazole and vinorelbine, respectively. Jurkat cells were previously known to respond to MT inhibitors and were therefore used as control. [file Image_1.TIF]

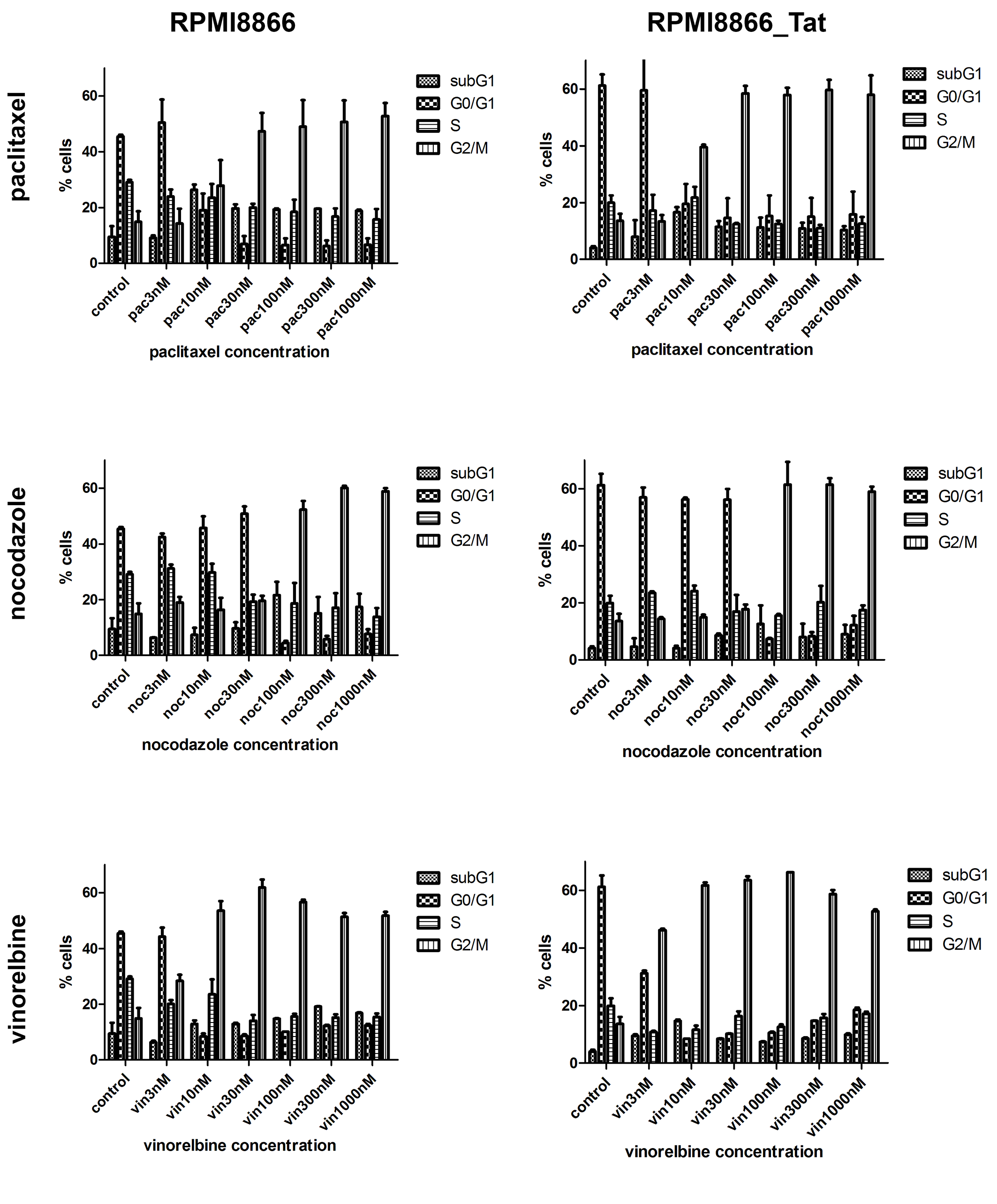

Supplement: FIGURE S2 — Lymphocytes treated with MT inhibitors (concentration range 3, 10, 30, 100, 300, and 1000 nM) for 24 h and stained for DNA content with Hoechst33342. Data from biological repeats on sub-G1, G0/G1, S, and G2/M population distributions presented as mean percentages with SD on a histogram. Graphs in columns represent RPMI8866 B-lymphocytes and RPMI8866-Tat-GFP B-lymphocytes, respectively. Graphs in rows represent paclitaxel, nocodazole and vinorelbine, respectively. [file Image_2.TIF]

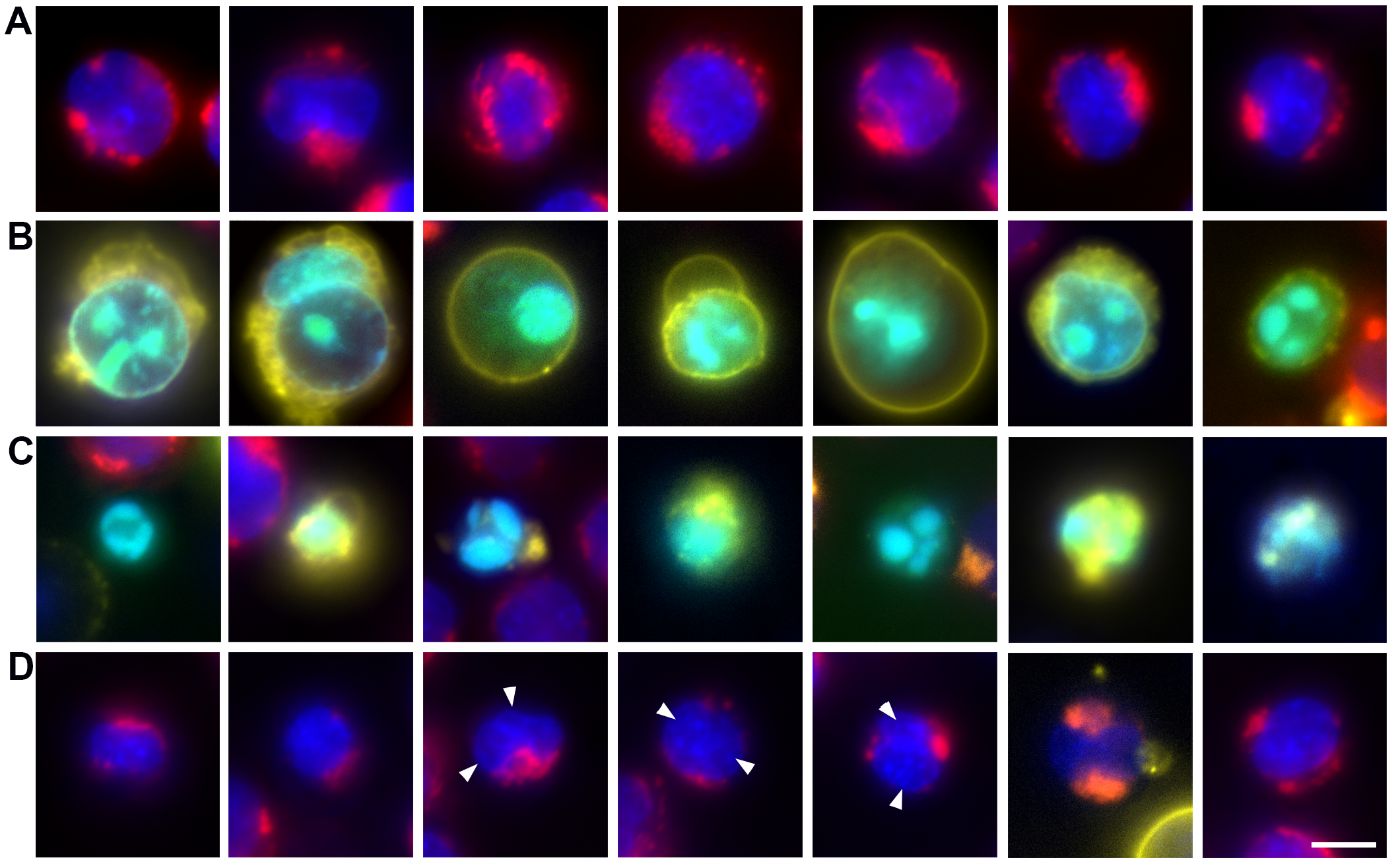

Supplement: FIGURE S3 — Fluorescence image galleries of RPMI8866 B-lymphocytes treated with a low dose of MT inhibitor 10 nM paclitaxel for 24 h. Simultaneous staining with Hoechst33342 (blue), TMRE (red), annexin V-Alexa 647 (yellow), and CellEvent (green). Scale bar – 10 μm. (A) Live cells with normal morphology have bright round nuclei, bright mitochondrial TMRE fluorescence and bear no apoptotic markers. (B) Apoptotic cells have TMRE-negative mitochondria, CellEvent caspase substrate staining co-localized with nuclear staining and surface-bound annexin V indicating phosphatidylserine externalization. (C) Cell debris and late apoptotic cells have smaller size, irregular shape, TMRE-negative mitochondria, deformed nuclei, often with CellEvent staining, and surface-bound annexin V indicating phosphatidylserine externalization. (D) small-sized cells with small nuclei, micronuclei, few TMRE-dim mitochondria, and no apoptotic markers. White arrowheads indicate micronuclei. [file Image_3.TIF]
